# New frontiers in oral submucous fibrosis: The promise of collagen based interventions

**DOI:** 10.6026/973206300214692

**Published:** 2025-12-15

**Authors:** Khushbu Lamba, Raj Kumar V, Priya Yadav, Rashika Jauhari, Sagar R Dixit, Aditi D Mugalikar

**Affiliations:** 1 Department of Oral and Maxillofacial Surgery, Jaipur Dental College, Jaipur, Rajasthan, India; 2 Department of Periodontics, ESIC Dental College and hospital, Rohini, Delhi, India; 3 Department of Conservative Dentistry and Endodontics, Jaipur Dental College, Jaipur, Rajasthan, India; 4 Department of Oral and Maxillofacial Surgery, YMT Dental College and hospital, Kharghar, Navi Mumbai, India

**Keywords:** Oral Sub-mucous Fibrosis, collagen, fibrosis management, collagen modulation

## Abstract

Oral Sub-mucous Fibrosis (OSMF) is a chronic, progressive and potentially malignant disorder characterized by inflammation and fibrosis of the oral mucosa, often leading to significant morbidity and impaired quality of life. The pathogenesis of OSMF is strongly associated with abnormal collagen metabolism, resulting in excessive deposition and reduced degradation of collagen fibres. Traditional therapeutic modalities have shown limited success in reversing the fibrotic changes, underscoring the need for novel treatment approaches. Therefore, it is of interest to explore the emerging role of collagen-based interventions including modulation therapies, enzymatic degradation, and regenerative techniques) which target key molecular pathways to improve functional outcomes and arrest disease progression. The potential of combining collagen- based approaches with regenerative and personalized medicine in the effective management of OSMF is also promising.

## Background:

The condition develops slowly and is considered insidious because its early symptoms may be mild or overlooked, but over time, the fibrosis becomes irreversible and debilitating [1]. OSMF is a serious public health concern because of its high prevalence in certain populations and its potential to transform into oral cancer, particularly squamous cell carcinoma [2]. Recently, researchers have begun focusing on therapies that target the underlying collagen metabolism to prevent or reduce fibrosis, offering new hope for more effective treatment options [3, 4-5].

## Pathophysiology of oral sub-mucous fibrosis:

The pathophysiology of Oral Sub-mucous Fibrosis (OSMF) involves a complex interplay of cellular, molecular and biochemical changes, primarily driven by chronic exposure to areca nut and its constituents. The key pathological process is abnormal collagen metabolism, where there is an imbalance between collagen synthesis and degradation, leading to excessive deposition of dense collagen fibers in the sub-mucosal connective tissues of the oral cavity. Areca nut contains alkaloids (such as arecoline) and tannins that stimulate fibroblasts to proliferate and increase collagen production. Simultaneously, these compounds inhibit the activity of collagenase enzymes responsible for collagen breakdown, further promoting accumulation [6]. Moreover, the reactive oxygen species (ROS) and inflammatory cytokines released during chronic exposure induce oxidative stress and promote tissue damage and fibrosis. These biochemical changes are accompanied by epithelial atrophy, resulting in thinner mucosa and increased vulnerability to trauma and malignant transformation. The chronic inflammation and fibrosis also lead to narrowing of the blood vessels (vascular constriction), reducing tissue perfusion, and contributing to tissue hypoxia, which further stimulates fibrosis [7]. Over time, the progressive stiffening of tissues results in reduced mouth opening (trismus), loss of tongue mobility and decreased elasticity of the oral mucosa. Advanced stages may involve the pharynx and esophagus, causing dysphagia. Thus, the pathophysiology of OSMF is characterized by a self-perpetuating cycle of collagen overproduction, impaired degradation, chronic inflammation, and tissue hypoxia, ultimately leading to irreversible fibrotic changes and functional impairment [8].

## Current management strategies for OSMF:

The current management strategies for Oral Sub-mucous Fibrosis (OSMF) aim to relieve symptoms halt disease progression and improve the patient's quality of life, although a complete cure remains elusive due to the irreversible nature of fibrosis. The cornerstone of management is the cessation of causative habits, particularly areca nut and tobacco chewing, which is critical to prevent further damage [9]. Patient counseling and behavioral interventions are essential to ensure compliance. In early stages, nutritional supplementation with vitamins (A, B-complex, C and E), iron and antioxidants helps counteract the oxidative stress and epithelial atrophy associated with the disease [10]. Pharmacological therapy includes the use of intralesional corticosteroids, which reduce inflammation and fibroblast proliferation and hyaluronidase, an enzyme that breaks down hyaluronic acid and helps reduce tissue stiffness. In some cases, a combination of both is injected into the fibrotic bands for better outcomes. Other agents like placental extracts, interferon-gamma, pentoxifylline and lycopene have been explored for their anti-inflammatory and antifibrotic effects [11]. Physiotherapy, involving mouth-opening exercises using devices like mouth gags or ice cream sticks, plays a supportive role in maintaining or improving jaw mobility. In advanced cases with severe trismus and dense fibrotic bands, surgical intervention may be required, such as the excision of fibrotic tissue followed by reconstruction using skin grafts or flaps like the buccal fat pad or nasolabial flap [12]. Laser surgery is also gaining popularity due to its precision and reduced healing time. Despite these interventions, recurrence and limited functional recovery remain concerns. Emerging therapies are now focusing on molecular targets, aiming to modulate collagen synthesis and degrade excess extracellular matrix components, providing hope for more definitive treatment in the future. Multidisciplinary care involving dentists, oral surgeons, nutritionists, and behavioral therapists is often necessary to manage the multifactorial nature of OSMF effectively [13] (Table 1 - see PDF).

## Collagen in OSMF:

Collagen plays a central role in the pathogenesis of Oral Sub-mucous Fibrosis (OSMF), as the disease is primarily characterized by the excessive deposition of collagen in the sub-mucosal connective tissues of the oral cavity. Under normal physiological conditions, collagen provides structural integrity and elasticity to oral tissues and its synthesis and degradation are tightly regulated [14]. However, in OSMF, this balance is disrupted due to the stimulatory effects of areca nut constituents, particularly arecoline, which enhance fibroblast activity and lead to increased collagen production. At the same time, the activity of matrix metalloproteinases (MMPs), the enzymes responsible for collagen degradation, is inhibited, often due to upregulation of their natural inhibitors known as tissue inhibitors of metalloproteinases (TIMPs). This results in the accumulation of dense, inelastic collagen fibers in the lamina propria and deeper connective tissues [15]. Histologically, there is a marked increase in Type I and Type III collagen, with changes in fiber orientation and cross- linking that contributes to tissue stiffness and reduced elasticity. The excessive and disorganized collagen not only causes the classic symptoms of OSMF, such as reduced mouth opening and mucosal rigidity, but also compromises local vascularity, leading to hypoxia and further fibrosis [16]. The role of collagen in OSMF has made it a critical target for therapeutic interventions, with current research exploring ways to inhibit its overproduction or enhance its degradation. Agents such as collagenase enzymes, antifibrotic drugs and molecular modulators of fibroblast function are being studied to restore the normal collagen turnover and potentially reverse or mitigate the fibrotic process. Therefore, understanding collagen dynamics is essential for both the diagnosis and management of OSMF [17]. Collagen-based therapeutic approaches: Collagen-based therapeutic approaches in Oral Sub-mucous Fibrosis (OSMF) are emerging as promising strategies aimed at addressing the core pathological feature of the disease- excessive collagen deposition. Traditional treatments have focused on symptomatic relief, but recent research emphasizes targeting the dysregulated collagen metabolism that underlies fibrosis [18]. One major approach involves the use of collagenase enzymes, which are capable of breaking down accumulated collagen fibers and restoring tissue flexibility. These enzymes help reduce the density and stiffness of fibrotic bands, thereby improving mouth opening and overall oral function. Another strategy includes the use of agents that inhibit fibroblast proliferation and collagen synthesis, such as interferon-gamma, which modulates the immune response and downregulates genes involved in collagen production [19]. Anti-fibrotic drugs like pentoxifylline and pirfenidone have shown promise by improving microcirculation, reducing inflammation and interfering with collagen formation pathways. Additionally, natural compounds such as curcumin, lycopene and aloe vera possess antioxidant and anti-inflammatory properties that indirectly affect collagen turnover by reducing oxidative stress and inhibiting the activation of fibroblasts [20]. Some therapies also focus on modulating the activity of matrix metalloproteinases (MMPs), the enzymes responsible for collagen degradation. Enhancing MMP activity or suppressing their inhibitors (TIMPs) may help restore the balance between collagen synthesis and breakdown. Furthermore, stem cell therapy and gene therapy are being explored to regenerate normal tissue and regulate collagen metabolism at the molecular level. Although many of these approaches are still in experimental or early clinical trial stages, they represent a shift toward more targeted, disease-modifying therapies for OSMF. By directly influencing the collagen pathway, these treatments hold the potential not only to alleviate symptoms but also to reverse or significantly slow disease progression [21].

## Recent advances and research trends:

In recent years, the research landscape surrounding OSMF has witnessed a paradigm shift towards understanding its molecular pathogenesis and exploring regenerative strategies. One of the most promising frontiers in this regard is the application of collagen-based interventions. Emerging studies have highlighted the central role of collagen metabolism in OSMF pathogenesis, were increased deposition and reduced degradation of collagen fibers lead to mucosal rigidity. This insight has paved the way for collagen-targeted therapies, including the use of collagenase enzymes, which help break down excessive collagen accumulation, thereby improving tissue flexibility. Moreover, collagen-based scaffolds and biomaterials are gaining momentum in regenerative medicine for their biocompatibility, low immunogenicity, and ability to support cell proliferation and angiogenesis. These materials can serve as carriers for controlled drug delivery or as structural frameworks for tissue regeneration. Bioengineered collagen matrices, when used in combination with growth factors or stem cells, have shown potential to reverse fibrosis and enhance mucosal healing in preclinical studies [22]. Recent advances in nanotechnology have further refined collagen delivery systems, enabling sustained release and targeted action at the fibrotic sites. Additionally, the integration of collagen-modulating agents such as curcumin, pentoxifylline and hyaluronidase into treatment regimens has shown synergistic benefits. Research is also delving into gene therapy approaches that can regulate collagen synthesis at the genetic level, potentially halting or reversing the progression of OSMF. Overall, collagen-based interventions represent a transformative approach that not only targets the symptomatic relief of OSMF but also addresses its root pathophysiology. With continued interdisciplinary research, these novel therapies hold immense promise in improving patient outcomes and reducing the malignant transformation potential of this debilitating condition [23].

## Challenges and future directions:

Despite the promising advancements in collagen-based interventions for Oral Sub-mucous Fibrosis (OSMF), several challenges continue to hinder their widespread clinical application. One of the foremost obstacles is the heterogeneity of the disease, with varying stages of fibrosis and symptom severity among patients, making it difficult to develop a one-size-fits-all treatment. Additionally, collagen-targeted therapies, such as enzymatic degradation or scaffold implantation, require precise delivery mechanisms to avoid unintended tissue damage or immune responses. The lack of standardized protocols for dosage, application frequency and long-term safety further complicates their integration into routine clinical practice. Moreover, most of the current evidence supporting these therapies stems from preclinical studies or small scale clinical trials, which limit their generalizability and regulatory approval. There is also the issue of cost and accessibility, especially in resource- limited settings where OSMF is most prevalent. Advanced biomaterials and nanotechnology- based delivery systems, though effective, may not be economically feasible for large-scale use in these populations [24]. Looking ahead, the future directions for collagen-based interventions in OSMF lie in developing personalized medicine approaches that tailor treatment to individual patient profiles based on genetic, molecular, and clinical markers. Larger multicentric clinical trials are essential to validate the safety, efficacy, and long-term outcomes of these interventions. Integration of artificial intelligence and imaging technologies could also aid in early diagnosis and monitoring treatment response more accurately. Additionally, combination therapies, involving collagen-based scaffolds with antifibrotic drugs, growth factors, or stem cells, are likely to yield synergistic effects and offer a more comprehensive therapeutic strategy. Collaborative efforts between researchers, clinicians and bioengineers will be crucial in refining these technologies and making them clinically and economically viable. Ultimately, a multidisciplinary approach that bridges basic science, material innovation and clinical application will be key to overcoming current limitations and ushering in a new era of effective, regenerative therapies for OSMF (Table 2 - see PDF).

## Discussion:

Oral Sub-mucous Fibrosis (OSMF) presents a significant clinical challenge due to its chronic, progressive, and irreversible fibrotic nature, often culminating in reduced mouth opening, burning sensation, dysphagia, and a high risk of malignant transformation. Traditionally, management strategies have focused on symptomatic relief using anti-inflammatory agents, corticosteroids, surgical release, and physiotherapy. However, these treatments offer limited success in reversing the fibrosis or restoring normal oral function. In light of these limitations, recent research has shifted its focus to the molecular underpinnings of fibrosis, particularly the role of collagen metabolism, opening new avenues for more targeted and regenerative treatment strategies. One of the most promising developments in this context is the exploration of collagen-based interventions. Collagen, being the primary extracellular matrix protein affected in OSMF, becomes excessively deposited and inadequately degraded, leading to tissue stiffness and functional impairment. This makes collagen both a biomarker and a therapeutic target. Advances in biotechnology and tissue engineering have led to the development of bioengineered collagen scaffolds, injectable collagenase enzymes and nanoparticle-based collagen delivery systems that aim to regulate this imbalance. These interventions not only modulate the deposition and breakdown of collagen but also serve as platforms for delivering anti-fibrotic agents or promoting tissue regeneration. Furthermore, studies involving collagen-based composites combined with stem cells, growth factors (such as bFGF and TGF-β modulators), or herbal antifibrotic compounds (like curcumin and aloe vera) have shown encouraging results in preclinical models. These approaches aim to enhance mucosal healing, reduce fibrosis, and restore oral function. Collagen matrices have also shown promise in acting as scaffolds for epithelial regeneration, indicating their potential utility in reversing the histopathological changes associated with OSMF. Nevertheless, the application of collagen-based therapies is still in its early stages. Most of the evidence supporting their efficacy is currently limited to laboratory studies or small clinical trials. To fully realize their potential, there is a pressing need for well-designed, large- scale clinical trials and long-term follow-up studies to assess their safety, efficacy and cost effectiveness. Moreover, optimizing the delivery systems, minimizing immunogenicity and making these treatments affordable and accessible in high-risk populations are crucial future goals [25].

## Conclusion:

Collagen plays a central role in the pathogenesis and progression of oral sub-mucous fibrosis. Targeting collagen metabolism offers a promising frontier in the management of this debilitating condition. Emerging therapies focusing on collagen modulation and regenerative medicine hold the potential to not only alleviate symptoms but also reverse fibrotic changes. Continued research and clinical innovation are essential to translate these advances into effective, patient-centered treatments.
